# *Saprochaete clavata* Invasive Infections – A New Threat to Hematological-Oncological Patients

**DOI:** 10.3389/fmicb.2019.02196

**Published:** 2019-10-29

**Authors:** Vladimir Buchta, Radka Bolehovská, Eva Hovorková, Oliver A. Cornely, Danila Seidel, Pavel Žák

**Affiliations:** ^1^Department of Clinical Microbiology, Faculty of Medicine in Hradec Králové, Faculty Hospital in Hradec Králové, Charles University in Prague, Hradec Králové, Czechia; ^2^Department of Clinical Biochemistry and Diagnostics, Faculty of Medicine in Hradec Králové, Faculty Hospital in Hradec Králové, Charles University in Prague, Hradec Králové, Czechia; ^3^The Fingerland Department of Pathology, Faculty of Medicine in Hradec Králové, Faculty Hospital in Hradec Králové, Charles University in Prague, Hradec Králové, Czechia; ^4^Cologne Excellence Cluster on Cellular Stress Responses in Aging-Associated Diseases (CECAD), University of Cologne, Cologne, Germany; ^5^Department I of Internal Medicine, ECMM Diamond Center of Excellence in Medical Mycology, German Centre for Infection Research (DZIF), University of Cologne, Cologne, Germany; ^6^Clinical Trials Centre Cologne (ZKS Köln), University of Cologne, Cologne, Germany; ^7^Department of Internal Medicine, ECMM Diamond Center of Excellence in Medical Mycology, University of Cologne, Cologne, Germany; ^8^4th Department of Internal Medicine – Division of Hematology, Faculty of Medicine in Hradec Králové, Faculty Hospital in Hradec Králové, Charles University in Prague, Hradec Králové, Czechia

**Keywords:** *Saprochaete*, *Magnusiomyces*, *Geotrichum*, leukemia, fungemia, diagnosis, therapy, FungiScope^TM^

## Abstract

**Background:**

*Saprochaete clavata* (formerly *Geotrichum clavatum*, now proposed as *Magnusiomyces clavatus*) is a filamentous yeast-like fungus that has recently been described as an emerging pathogen mostly in patients with acute leukemia.

**Methods:**

This is a retrospective study of patients diagnosed with proven and probable *S. clavata* infection at the University Hospital, Hradec Králové, Czechia between March 2005 and December 2017. Previous cases were identified from the literature and FungiScope^®^ database.

**Results:**

Six new cases (5 females, 1 male) of blood-stream *S. clavata* infections at the hemato-oncological department were described including epidemiological data of additional 48 patients colonized with the species. Overall, 116 strains of *S. clavata* were isolated from different clinical specimens of 54 patients; most of them belonged to the respiratory tract (60.3%). *S. clavata* was the most frequent species among arthroconidial yeasts (*Trichosporon*, *Galactomyces*, *Magnusiomyces*) recovered from the blood. All our patients with *S. clavata* infection had profound neutropenia, a central venous catheter, broad-spectrum antibiotics and antifungal prophylaxis; four had a history of a biliary tract system disease. The diagnosis was based on a positive blood culture in all patients. Four patients died of multiorgan failure and sepsis despite treatment with lipid-based amphotericin B and/or voriconazole. From the literature and FungiScope database, 67 previous cases of *S. clavata* infections were evaluated in context of our cases.

**Conclusion:**

*Saprochaete clavata* infection represents a life-threatening mycosis in severely immunocompromised patients. The successful outcome of treatment seems to be critically dependent on the early diagnosis and the recovery of underlying conditions associated with immune dysfunction or deficiency.

## Introduction

Invasive systemic infections caused by fungi have increasingly been recognized and represent relevant cause of mortality and morbidity in growing segment of immunocompromised patients for the last decades (Miceli et al., 2011; Armstrong-James et al., 2017). The predisposing conditions are largely associated with changing spectrum of patients (age structure, co-morbidities) who are associated with a more risky therapeutic management such as an extensive surgery or aggressive treatment modalities. The majority of these risk factors are related to impaired immune defense mechanisms (hematological malignancies and transplantations, neutropenia, immunodeficiency, HIV), often as a result of the use of immunosuppressant drugs (e.g., corticosteroids, cyclosporine, biologics), the disruption of skin and mucosa integrity (extensive surgery, catheterization, burns, mucositis), and interference of antibiotics with the indigenous microbiota (dysbiosis) (Gulcan et al., 2016; Vallabhaneni and Chiller, 2016; Vallabhaneni et al., 2016; Colombo et al., 2017). These conditions make patient population vulnerable to opportunistic pathogens including fungi such as *Aspergillus*, *Candida*, *Cryptococcus* or Mucorales (Vallabhaneni et al., 2016, 2017; Colombo et al., 2017). Apart from the main fungal etiology, there is a rare and taxonomically diverse group of opportunistic yeasts belonging to the genera *Galactomyces*, *Trichosporon*, and *Magnusiomyces* (*Saprochaete*), which share morphological characteristics, namely the production of arthroconidia (Hazen, 1995; Henrich et al., 2009; Repetto et al., 2012; Meletiadis and Roilides, 2013; Arendrup et al., 2014; Durán Graeff et al., 2017; Fernández-Ruiz et al., 2017). Most systemic infections caused by those arthroconidial fungi are attributable to two species, *Magnusiomyces capitatus* (synonym *Saprochaete capitata*) and *Trichosporon asahii*. *Saprochaete clavata* has emerged as a new pathogen in hematological patients in French and Italian hospitals (Lacroix et al., 2007; Camus et al., 2014; Picard et al., 2014; Vaux et al., 2014; Cornely et al., 2015; Del Principe et al., 2016; Favre et al., 2016; Esposto et al., 2018; Leoni et al., 2018). Taxonomy studies showed that *S. clavata* and *M. capitatus* are closely related (de Hoog et al., 1986; Guého et al., 1987; Smith and Poot, 1998). Today, three main clades of the arthroconidial genera are discriminated: *Galactomyces* and *Dipodascus* which are associated with the *Geotrichum* anamorphs, while *Magnusiomyces* with the *Saprochaete* species (De Hoog and Smith, 2004; Daniel et al., 2014). Recently, owing to the principle the one fungus, one name, dual naming has been replaced and *M. capitatus* (synonym *S. capitata, Dipodascus capitatus*) and *S. clavata* are now accepted (De Hoog and Smith, 2004; Hawksworth et al., 2011). In addition, Kaplan et al. (2017) have pointed out that the rules of nomenclature using the oldest valid name and the molecular phylogeny would necessitate renaming *S. clavata* to *Magnusiomyces clavatus*. Majority of characteristics of epidemiology, diagnosis and therapy of *S. clavata* infections are similar to those caused by *M. capitatus* and *T. asahii* (Kaplan et al., 2017). They include frequent recovery from blood, lack of specific diagnostic methods, no specific breakpoints for antifungal susceptibility test results and no optimal therapeutic regimen. Moreover, epidemiological data are scarce; there are only a few details about source and transmission of *S. clavata*, although it has the potential to cause outbreaks (Bougnoux et al., 2018).

Here, we present six new cases of severe infection caused by *S. clavata* diagnosed in the hematologic intensive care unit and epidemiological data of hospital recordings of 48 patients colonized with the yeast at the University Hospital, Hradec Králové, Czechia between March 2005 and December 2017, which are discussed in context of other *S. clavata* cases reported in the literature and international registry FungiScope^®^.

## Materials and Methods

### Patient Information

Clinical data of patients with diagnosed *S. clavata* infection were collected including basic demographics, underlying diseases, clinical picture, antifungal therapy, and clinical outcome (Table 1). Cases with probable or proven infection classified according to the EORTC/MSG criteria were included (De Pauw et al., 2008). A literature search using PubMed for respective cases was done with the search terms “*Saprochaete*,” “*Geotrichum*,” “*Dipodascus*,” “*Magnusiomyces*,” “fungemia,” “invasive infection,” and “rare mycoses.” In addition, cases identified from the FungiScope^®^ registry were selected (Seidel et al., 2017).

**TABLE 1:** Baseline characteristics of Czech patients with *Saprochaete clavata* infection.

	**Patient 1**	**Patient 2**	**Patient 3**	**Patient 4**	**Patient 5**	**Patient 6**
Sex	Male	Female	Female	Female	Female	Female
Age	45	61	63	58	50	66
Underlying present disease	AML – late relapse	AML – new	AML – new	AML – early relapse	AML – late relapse	DLBCL Bile duct obstruction Hemorrhagic shock *Candida glabrata* septic shock
Previous diseases/Risk factors	AML (alloHSCT) Acute GvHD Cholelithiasis Cholecystectomy Common bile duct obstruction (internal biliary drainage) HSV infection	Cholelithiasis Cholecystectomy Chronic pancreatitis Ovarial cystadenofibroma (adnexectomy)	Cholelithiasis Cholecystectomy	AML (1st alloHSCT) Acute GvHD Cervix ca *in situ* (hysterectomy) Renal ca (resection) Colorectal ca (resection, radio) Chronical anal fissura Recurrent CDI MDS–EB2 (Aza)	HSV myocarditis AML (autoHSCT)	Cholelithiasis Cholecystectomy
Chemotherapy regimen	Ida-HiDAraC	Chemotherapy 3 + 7 FLAG-Ida	1st course Chemotherapy 3 + 7 2nd course of HiDAC	FLAG-Ida TBI 3Gy + F and 2nd alloHSCT	Chemotherapy 3 + 7	SoluMedrol and R-CHOP, intrathecal (hydrocortisone, MTX + AraC)
**Neutropenia (days)**						
ANC < 100/ml	19	45	5	20	20	8
ANC 100–500/ml	0	0	7	3	0	0
Duration neutropenia (days) ANC <100/ml at the time positive culture	14	33	7	12	8	3
Diabetes mellitus	No	No	No	Yes	No	No
Mucositis	Yes, grade III	Yes, grade III	Yes, grade II	Yes, grade IV	Yes, grade II	No
CVC	Yes	Yes	Yes	Yes	Yes	Yes
Urinary catheter	Yes	Yes	No	Yes	Yes	Yes
Nasogastric tube	Yes	Yes	No	Yes	Yes	Yes
Pulmonary ventilation	Yes	Yes	No	No	No	Yes
Parenteral nutrition	Yes	Yes	No	Yes	Yes	Yes
**Prophylaxis**						
Antibiotic	Ciprofloxacin	Ciprofloxacin	Ciprofloxacin	Ciprofloxacin	No	Ciprofloxacin
Antiviral	Acyclovir	Acyclovir	No	Acyclovir	Acyclovir	No
Antifungal	Fluconazole	Fluconazole	Fluconazole	Voriconazole	Fluconazole	Fluconazole
Antibiotic therapy	Meropenem	Teicoplanin	Ciprofloxacin	Linezolid	Cephoperazone	Piperacillin/Taz
	Vancomycin	Imipenem	Piperacillin/Taz	Meropenem	Meropenem	Meropenem Linezolid
	Teicoplanin	Cephoperazone	Metronidazole	Levofloxacin	Teicoplanin	Vancomycin
		Piperacillin/Taz			Cefepime	Amikacin
		Vancomycin				
		Amikacin				
Antifungal therapy (*S. clavata* infection)	Amphotericin B (1 mg/kg qD)	Amphotericin B (1 mg/kg qD) Lipid-based AMB (Abelcet 5 mg/kg qD)	Lipid-based AMB (Abelcet 5 mg/kg qD) Voriconazole (200 mg i.v. q12 h)	Amphotericin B (0.7–1 mg/kg qD) Lipid-based AMB (Abelcet 5 mg/kg qD) Voriconazole (200 mg p.o. q12 h)	Amphotericin B (0.7–1 mg/kg qD.)	Micafungin (100 mg qD) Voriconazole (200 mg i.v. q12 h)
Antifungal susceptibility	E: AMB 1; FLZ 12; VRZ 3; PSZ 8; CFGN 32	E: AMB 2; FLZ 128; ITZ 4; VRZ 8; PSZ 32; CFGN 32	M: AMB 0.5; FLZ 8; ITZ 0.25; VRZ 0.094; PSZ 0.5; AFGN 2; CFGN 8; MFGN 2	M: AMB 0.5; FLZ 48; ITZ 0.25; VRZ 1; PSZ 1; AFGN 16; CFGN 16; MFGN 2; 5FC 0.12	D: AMB S; FLZ S; ITZ S; KTZ S; 5FC S	M: AMB 1; FLZ 4; ITZ 0.125; VRZ 0.03; PSZ 0.25; AFGN 0.5; CFGN 1; MFGN 0.5; 5FC 0.25
Outcome Cause of death	Died septic shock, MODS	Died progression AML septic shock, MODS brain edema	Survived	Died septic shock, MODS	Died septic shock, MODS	Died septic shock, MODS
**Culture-positivity for *S. clavata***						
Blood/Other	Yes, 3×/Autopsy (kidney)	Yes, 3×/No	Yes, 1×/No	Yes, 3×/Rectum 2×	Yes, 2×/Autopsy (lungs) Wound swab	Yes, 2×/Urine Bile

*5FC – flucytosine, auto/alloHSCT – autologous/allogeneic hematopoietic stem cell transplantation, AFGN – anidulafungin, AMB – amphotericin B, AML – acute myeloid leukemia, ANC – absolute neutrophil count, CDI – *Clostridium difficile* infection, CFGN – caspofungin, D – disk test (mm), E – Etest (MIC, mg/l), FLZ – fluconazole, GvHD – graft vs. host disease, HSV – herpes simplex virus, ITZ – itraconazole, M – MIC, mg/l (broth dilution), MFGN – micafungin, MODS – multiple organ dysfunction syndrome, PSZ – posaconazole, S – susceptible category (disk test), Taz – tazobactam, VRZ – voriconazole.*

### Collection and Identification of Fungal Isolates

**FIGURE 1:**
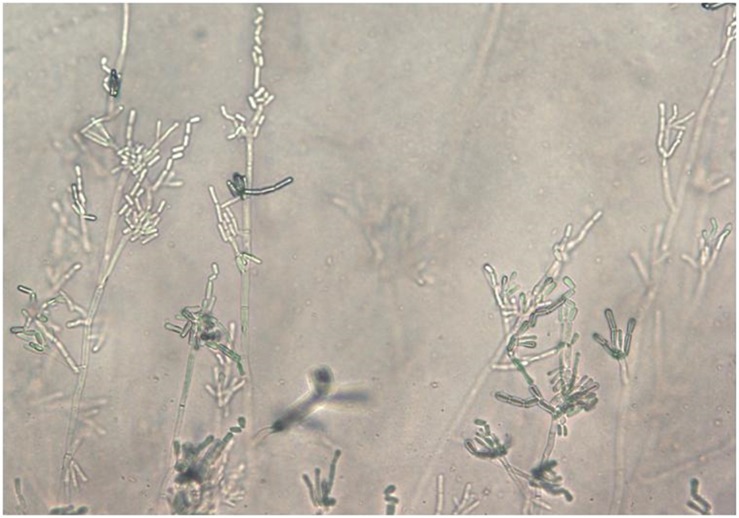
*Saprochaete clavata* arthroconidia on Corn-Meal agar after 5 days at 35°C (slide culture; magn. ×40).

All clinical specimens – cerebrospinal fluid, bronchoalveolar lavage (BAL) fluid, sputum, tracheal aspirate, urine, stool, wound swab, cervicovaginal fluid, punctate, skin adnexa, upper respiratory tract samples – obtained from patients hospitalized in University Hospital were routinely analyzed in mycological laboratory by inoculating onto mycological agar (SDA) to get individual colonies for further investigation such as biochemical tests (biochemical profile assessment), additional cultivation on Corn-meal agar (description of fungal micromorphology), antifungal susceptibility testing. Most of the conventional diagnostic methods were replaced after availability MALDI TOF mass spectrometry (protein profile assessment). Blood samples were cultivated in the BACTEC system using Mycosis medium or media for aerobic bacteria (Beckton Dickinson Diagnostic Instrument System). In case of BAL fluid, sputum, tracheal aspirate, and urine the samples were quantified after inoculation on SDA by means of calibrating bacteriological loops. Fungal identification including *S. clavata* was based on a combination of microscopic examination of morphological traits on Corn-meal agar, especially arthrospore formation (Figure 1), colony appearance on chromogenic agar (Colorex, Trios, Czechia), and biochemical pattern methods based on the evaluation of urease production, in-house carbon auxanogram assimilation tests (17 carbohydrates and sugar alcohols) (sugar disks provided by ITEST plus, Czechia), and/or using of the API ID32C test (BioMérieux, Czechia). Three blood isolates were additionally identified by MALDI-TOF mass spectrometry (Bruker).

Criteria for *S. clavata* identification included the formation of arthrospores, the absence of urease production, and assimilation of glucose, cellobiose, variable galactose, and negative xylose. These physiological characteristics seem to be sufficient for routine laboratory discrimination of the main arthroconidial fungi pathogenic for humans, especially between *M. capitatus* and *S. clavata* (Smith and Poot, 1998; Kaplan et al., 2017).

Isolates from Patient 3 and 4 were analyzed by sequencing. DNA was extracted from the strains using a QIAamp^®^ DNA Mini Kit (Qiagen) protocol and the 18S rRNA gene was amplified using PCR (Millar et al., 2000). Sequences were analyzed using BLAST at NCBI^1^.

### Antifungal Susceptibility Testing

The minimum inhibitory concentration (MIC) was determined using Etest (BioMérieux, Czechia) or Sensititre YeastOne (Trek Diagnostics, BioVendor, Czechia) following the instructions of the manufacturer. Sabouraud dextrose agar (BioMérieux CZ) and Mueller-Hinton agar with 2% glucose (LabMediaServis, Czechia) were culture media for disk test in the period of 1995 to 2005 and 2006 to 2017, respectively (CLSI, 2009). The latter agar was also used in the Etest. Since 2016 the paper disks in agar diffusion method has been replaced with tablets (Neo-Sensitabs, Rosco Diagnostica), but this modification of methodology concerned only two of 55 *S. clavata* isolates tested. All strains were included in the statistical analysis according to the following criteria: one isolate (of the same species) per material and per one patient. Quality control strains of *Candida albicans* ATCC 90028, *Candida krusei* ATCC 6258 and *Candida parapsilosis* ATCC 22019 were included.

### Epidemiological Investigation

The incidence of *S. clavata* strains at the University Hospital Hradec Králové during the period of 1995–2017 was retrospectively evaluated based on the recordings of the laboratory information system and the criteria mentioned above. Blood, cerebrospinal fluid, BAL fluid, sputum, tracheal aspirate, urine, and other clinical specimens were microbiologically investigated.

### Cases

**FIGURE 2:**
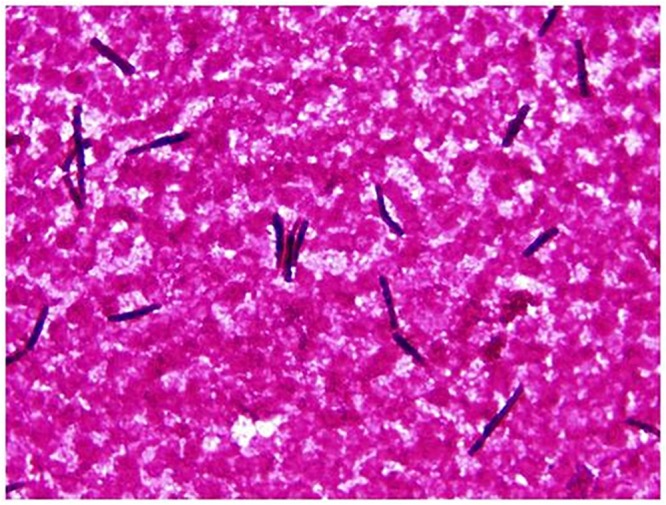
*Saprochaete clavata* in blood culture. Arthroconidia-like hyphal fragments (Gram staining, magn. ×1000).

**FIGURE 3:**
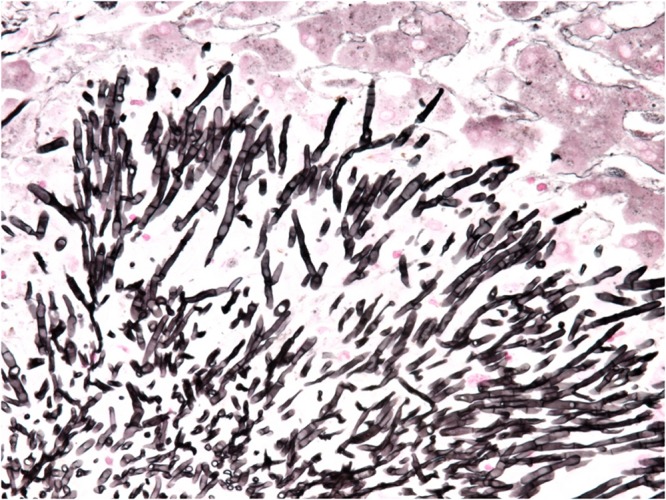
Detail of microcolony of *Saprochaete clavata* invading liver by septated hyphae branching in acute angle (methenamine silver stain, magn. ×400).

**FIGURE 4:**
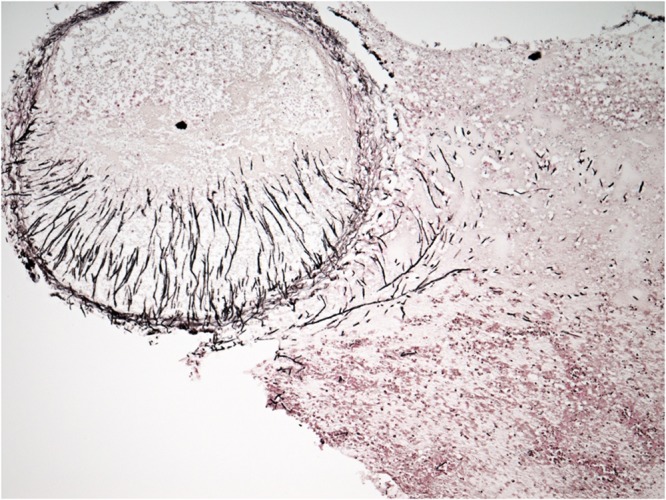
Angioinvasion of spinal cord with hyphae (methenamine silver stain, magn. ×100).

Six patients were diagnosed with an infection due to *S. clavata* in the hematologic intensive care unit at our University Hospital between 2005 and 2017 (Table 1). The median age was 50.5 years (range 45 to 66 years), five patients (83.3%) were female. Five patients were treated for acute myeloid leukemia (AML) and one for diffuse large B-cell lymphoma (DLBCL). The *S. clavata* infection in all patients was diagnosed based on a positive blood culture (Figure 2). In all patients, their management was complicated by bacterial opportunistic infections and by intensive therapy with broad-spectrum antibiotics and anticancer drugs including cytarabine. Five patients developed septic shock and required the use of artificial ventilation and/or hemodialysis. Histological investigation of necroptic samples demonstrated angioinvasivity of vessels with the tendency to disseminate to various organs, including the peritoneum, liver or spinal cord. Methenamine silver staining showed septate hyphae branching in acute angles unrecognizable from *Aspergillus* mycelium (Figures 3, 4). Four patients died of septic complications due to fungal and bacterial infections and concomitant hematologic disease. Two patients survived, but one died from an early relapse of AML later. Only one patient (no. 3) experienced a complete remission of AML. The relevant aspects of the treatment of individual patients are summarized in Table 1.

#### Patient 1

The male patient was diagnosed with AML 8 years after completing the treatment for Hodgkin lymphoma. The treatment of AML consisted of chemotherapy and allogeneic hematopoietic stem cell transplantation (HSCT), which was performed during complete remission. The patient’s condition was complicated by a biliary obstruction of unknown etiology and required external biliary drainage. The first relapse of AML occurred after 4 years. The second remission of AML was not achieved after induction chemotherapy. The patient developed fever 20 days after chemotherapy (FLAG-Ida). *S. clavata* was cultured from blood. The patient developed septic shock and died of multiple organ dysfunction syndrome (MODS) 43 days after diagnosing *S. clavata*.

#### Patient 2

The female patient previously underwent resection for ovarian cancer. AML was diagnosed 3 years after the completion of the cancer treatment. A complete remission of AML was induced only after a second course of induction chemotherapy. A blood culture was positive for *S. clavata*. During a prolonged pancytopenia (absolute neutrophil count below 100/ml lasted 45 days) septic shock and MODS developed 8 days after diagnosing *S. clavata*. After the completion of treatment irreversible brain damage resulted. Subsequently, the active treatment of AML was terminated and the patient died 3 months after the diagnosis of AML.

#### Patient 3

The female patient previously underwent surgical treatment for cholecystitis. Six years later, she was diagnosed with AML. A complete remission was induced with the first course of induction therapy. *S. clavata* infection occurred after a first course of consolidation chemotherapy with high-dose cytarabine. This is the only patient who did not develop a septic shock and recovered hematopoietic function. She is still alive and remains in complete remission 87 months after allogeneic HSCT.

#### Patient 4

The female patient previously underwent resection for renal cell carcinoma as well as a resection and radiotherapy for colorectal carcinoma. She continued to suffer from a chronic anal fistula and a recurring *Clostridium* infection. Three years after undergoing radiotherapy patient was diagnosed with myelodysplastic syndrome (MDS) which developed into AML. The leukemia was treated with chemotherapy and allogeneic HSCT (alloHSCT) using a reduced-intensity regimen. Three months after alloHSCT acute graft-versus-host disease (GvHD) affected the skin and later on the intestine as well. The GvHD resolved after adding corticosteroid therapy. At the same time, she presented the first early relapse of AML and a new course of induction therapy (FLAG-Ida) was performed. The patient achieved incomplete remission of AML. The following month she was admitted for gastrointestinal bleeding and paralytic ileus. Due to histological confirmation of acute intestinal GvHD, the patient received corticosteroids in addition to standard supportive therapy. The general condition of the patient was very good and without gastrointestinal GvHD manifestation. Later, after the second alloHSCT, *S. clavata* was isolated from blood and stool. The course of treatment was complicated with septic shock and MODS 7 days after diagnosis *S. clavata*, which ultimately led to death.

#### Patient 5

The patient was treated for AML with chemotherapy and autologous peripheral blood stem cell transplantation. Three years later, the patient had a relapse of AML. Group B streptococci and *C. albicans* were cultivated from the nasopharyngeal swab. During a period of deep neutropenia, blood culture was positive for *S. clavata*. Eventually she developed septic shock with MODS and that resulted in death.

#### Patient 6

The female patient was treated for DLBCL. Due to infiltration and subsequent external biliary obstruction with a lymphoma, external drainage had to be performed. Following a course of R-CHOP chemotherapy, she developed combined hemorrhagic and septic shock with MODS. After the patient was stabilized, a surgical review identified the origin of the hepatic bleeding and liver packing was provided. Another septic shock occurred 2 weeks after candidemia caused by *C. glabrata*, when blood cultures became positive for *S. clavata*. Afterward, septic shock and MODS developed and the patient died.

## Results

**FIGURE 5:**
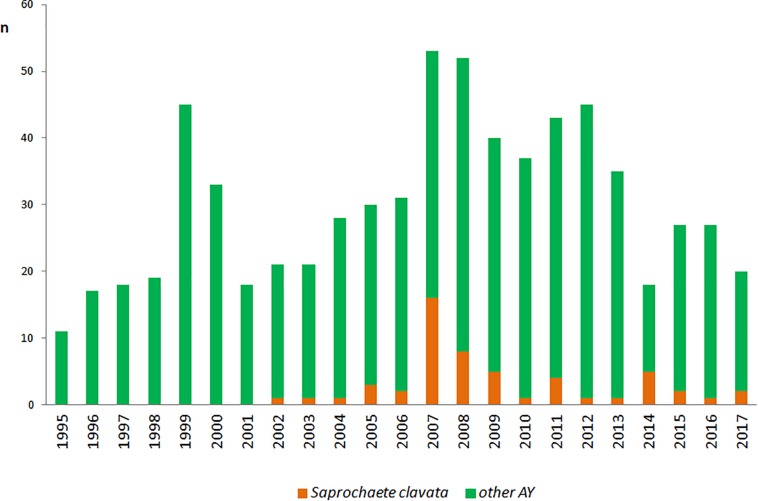
Incidence of *Saprochaete clavata* and arthroconidial fungi at the University Hospital Hradec Kralové. n – number of isolates (one isolate of a given species per one patient). Other AY – number of isolates of arthroconidial yeasts (*Galactomyces candidus, Magnusiomyces capitatus, Trichosporon asahii*) without *Saprochaete clavata.*

**FIGURE 6:**
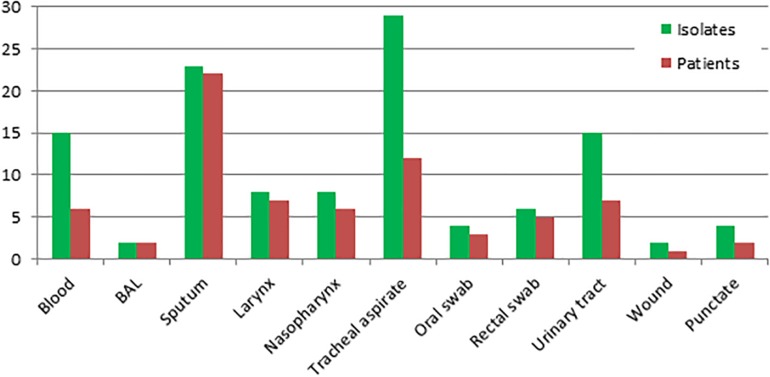
Distribution of *Saprochaete clavata* by clinical material at the University Hospital Hradec Kralové in 1995–2017. Isolates – number of *S. clavata* isolates (including repeated ones per material and per one patient). Patients – number of *S. clavata* strains per patient without repeated isolates from the same material. BAL – bronchoalveolar lavage fluid, TAS – tracheal aspirate.

Overall, 116 strains of *S. clavata* from 54 patients were obtained during the follow-up period. Almost all patients (*n* = 50, 92.6%) were colonized with the species, only six (11.1%) developed an infection with positive blood culture of which four had no other *S. clavata* findings and two were colonized – one before (biliary drainage fluid) and one after (rectal swab) fungemia. *S. clavata* was first identified in our institution in 2002 and was outnumbered by other arthroconidial species, especially *T. asahii* and *M. capitatus*, every year during the study period; only in 2007 it represented the most numerous species among these fungi (Figure 5). In contrast to other arthroconidial yeasts, female patients were more often colonized with *S. clavata* than males (55.6 vs. 44.4%). The distribution of culture positive findings suggested three main sources of *S. clavata* in the human body: respiratory tract and to a lesser extent, the urogenital tract and the gastrointestinal tract (Figure 6). These sources partially overlapped with colonization potential that can be expressed as repeated isolations from the same material. They are tracheal aspirate, urine samples, and punctate fluid, in which the number of isolates per material was more than doubled compared to other materials with usually one isolate per specimen (Figure 6). The exception was blood where four of six patients had repeated positive blood samples for *S. clavata*. In addition, *S. clavata* was the most common species among the arthroconidial yeasts isolated from the blood (6 × *S. clavata*, 3 × *M. capitatus*, and 2 × *T. asahii*) but there was no previous colonization of any catheter.

**FIGURE 7:**
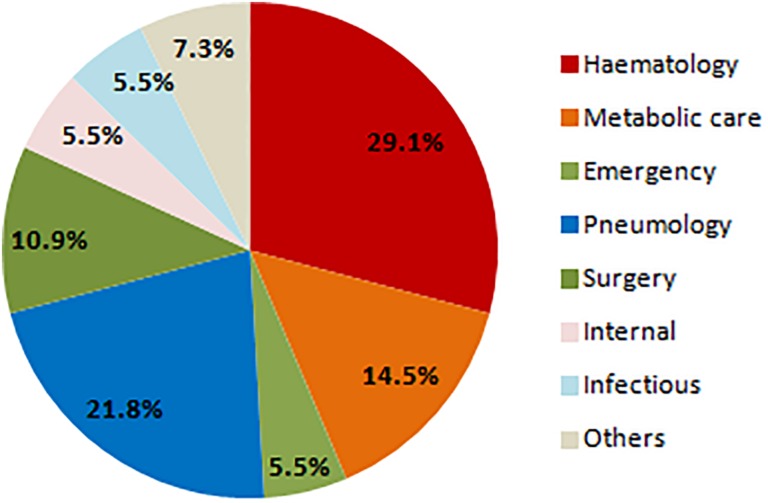
Distribution of *Saprochaete clavata* isolates by clinic at the University Hospital Hradec Králové in 1995–2017. n – number of isolates (one isolate of a given species per one patient).

Prevalence of *S. clavata* in ICU patients was similar to those from standard departments (50.8% in ICU vs. 49.2% in non-ICU), but all fungemic patients were hospitalized at the oncological-hematological department. Most of *S. clavata* isolates came from the patients of this clinic (29.1%), followed by pulmonary (21.8%) and geriatric-metabolic department (14.6%) (Figure 7).

Antifungal susceptibility testing was affected by the method used during study period as the individual methods changed (Tables 2, 3). In sum, 13 strains were tested for MICs (Etest, Sensititre YeastOne) and 73 strains for inhibition zones (agar diffusion test). Based on the criteria (see section “Materials and Methods”), 12 and 55 of the strains were included in the analysis, respectively (Tables 2, 3). In general, Etest provided higher MICs than broth dilution format (Sensititre YeastOne). Our *S. clavata* strains displayed relatively low MICs against amphotericin B, voriconazole, itraconazole, flucytosine, and partly posaconazole, while the MICs of fluconazole and echinocandins were high (Table 2). The results of the disk test varied greatly. For voriconazole, posaconazole, flucytosine and echinocandins MICs corresponded well with the results from the disk test (Tables 2, 3).

**TABLE 2:** Review of *in vitro* susceptibility of *Saprochaete clavata* isolates to antifungal drugs.

**Specimen (n)**	**Drug**	**AMB**	**AFGN**	**MFGN**	**CFGN**	**PSZ**	**VRZ**	**ITZ**	**FLZ**	**ISZ**	**5FC**	**Source**
												
		**GM**	**GM**	**GM**	**GM**	**GM**	**GM**	**GM**	**GM**	**GM**	**GM**	
	**Methods^∗^**	**Range**	**Range**	**Range**	**Range**	**Range**	**Range**	**Range**	**Range**	**Range**	**Range**	
Blood (7)	Etest	0.955	32	3	36.6	9.97	0.676	0.794	18.4			This study
Sputum (2)	n^#^	0.25–2	32	3	32–48	2–32	0.094–8	0.25–4	4–128			
Others^§^ (4)	YeastOne	0.574	1.74	2	8	0.285	0.058	0.092	6.96		0.091	
	n = 5	0.5–1	1–2	1–4	2–16	0.12–0.5	0.015–0.5	0.03–0.25	2–32		0.06–0.12	
Clinical isolates (4)	CLSI			2.25		4	0.19			0.88		Pfaller et al., 2015
	M27-A3			1–4		4	0.062–0.5			0.5–2		
Human/	CLSI	0.22	2	6.7		0.25	0.25	0.27	19	0.54		Kaplan et al., 2017
Dishwasher (8)	M27-A3	0.125–0.5	2	2–8		0.25	0.063–0.5	0.25–0.5	16–32	0.125–1		
Blood (3)	Etest	1–1.5	> 32	>32	> 32	0.19–0.5	0.094–0.125					Picard et al., 2014
Blood (3)	YeastOne	0.42	1	0.5	8	0.17	0.05	0.09	2.67			Del Principe et al., 2016
	Sensititre	0.25–0.5	1	0.5	8	0.125–0.25	0.03–0.06	0.03–0.12	2–4			
Blood (45)	EUCAST	0.5			8	0.5	1				0.25	Vaux et al., 2014
	E.Def 7.2	0.125–1			1–8	0.125–1	0.06–2				<0.125–1	
Blood (1)	Etest	1				0.75	0.064		12			Camus et al., 2014
Blood (1)	EUCAST	0.25		>4	>4	0.5	0.5		32		0.25	Favre et al., 2016
	E.Def 9.1											
Clinical isolates (4)	Etest	1.25	3	0.56	>32	0.16	0.13	0.13	20.3	0.014	16.1	Durán Graeff et al., 2017
		1–2	2–4	0.25–1	>32	0.032–0.25	0.016–0.25	0.002–0.25	1–32	0.004–0.25	0.06–>32	
Blood (1)	MIC test	≤0.5					0.125	≤0.125	2		≤4	Liu et al., 2018
Blood (18)	YeastOne	0.96				0.56	0.34	0.31	17.96	0.71	0.18	Esposto et al., 2018
	Sensititre	0.5–1				0.25–1	0.03–1	0.12–0.5	8–64	0.12–4	0.06–0.5	
Blood (1)	YeastOne	0.25	R	R	R	0.25	0.5		32		0.12	Salgüero Fernández et al., 2018
	Sensititre											

*^∗^Quantitative test (MIC, mg/ml); GM – geometric mean; n – number of isolates. ^#^The number of strains tested with Etest in brackets, AMB (7), VRZ (6), FLZ (5), PSZ (5), ITZ (3), CFGN (3), AFGN (1), MFGN (1). §Laryngeal swab, punctate, urine, drainage fluid. R – interpreted as resistant according to EUCAST standard (document not specified), AMB – amphotericin B, 5FC – flucytosine, AFGN – anidulafungin, MFGN – micafungin, CFGN – caspofungin, PSZ – posaconazole, VRZ – voriconazole, ITZ – itraconazole, FLZ – fluconazole, ISZ – isavuconazole.*

**TABLE 3:** Antifungal susceptibility of *Saprochaete clavata* isolates^∗^ by disk diffusion method at the University Hospital, Hradec Králové in the period of 1995–2017.

	**AMB**	**FLZ**	**ITZ**	**VRZ**	**PSZ**	**KTZ**	**CFGN**	**5FC**
n	60	69	68	53	5	16	5	7
GM	12.6	15.9	14.8	20.2	18.4	23.6	8.8	35.0
range	8–25	6–32	9–29	6–33	14–22	18–28	6–12	22–51
IZ50	13	18	14	20	19	25	9	35
IZ90	9.5	6	11	15		19		

*^∗^Only isolates that met the following criteria were included in the statistical analysis: one isolate/species per one material and per one patient. GM – geometric mean (inhibition zone in mm), n – number of strains, IZ50/IZ90 – lower limit of inhibition zone (mm) encompassing 50%/90% of isolates tested. AMB – amphotericin B, FLZ – fluconazole, ITZ – itraconazole, VRZ – voriconazole, PSZ – posaconazole, CFGN – caspofungin, 5FC – flucytosine, KTZ – ketoconazole.*

Review of the literature and FungiScope^®^ register revealed 73 cases of *S. clavata* infections in 10 countries most of which located in the Mediterranean (for details see Table 4). Only ten patients were from other regions – Germany, Serbia, China, and Czechia. The vast majority of patients manifested similar clinical signs and symptoms (neutropenia, fever, positivity of blood culture, dissemination and sepsis or septic shock, diarrhea) at time of diagnosis of *S. clavata* infection. The same was true for underlying conditions, including central venous catheter (CVC), broad-spectrum antibiotic therapy, aggressive chemotherapeutic regimens with cytarabine, and, in case of the French cohort, bacterial digestive decontamination (Vaux et al., 2014). Most patients were treated with voriconazole and/or lipid-based amphotericin B, but mortality rate was extremely high (>65%) (Table 4). *In vitro* and *in vivo* results confirmed that *S. clavata* is intrinsically resistant to echinocandins (Table 2).

**TABLE 4:** Summary of case characteristics of *Saprochaete clavata* infections from literature and FungiScope^®^.

**Study**	**Country**	**Sex**	**Age**	**Underlying disease**	**Risk factor^#^**	**Clinical form**	**Positive specimen**	**Lab diagnosis**	**Drug**	**Dosage**	**Duration**	**Outcome**
Lacroix et al., 2007	France	M	14	AML	CVC, cytarabine	Sepsis	Blood	Blood culture	E: AMB	ns	1 day	Survived
									T: L-AMB + VRZ		5 days	
									T: VRZ + 5FC		ns	
		
		M	59	AML	CVC, cytarabine	BSI	Blood, urine,	Blood culture,	P: CFGN	ns	7 days	Survived
							biopsy (skin)	GM negative	E: CFGN + L-AMB	3 mg/kg/d (L-AMB)	7 days	
									E: L-AMB + PSZ		4 days	
									T: L-AMB + 5FC + PSZ	5 mg/kg/d (L-AMB)	7 days	
							urine		T: L-AMB + 5FC + VRZ		21 days	

Picard et al., 2014	France	F	46	AML	CVC, cytarabine, digestive decontamination (GEN, COL), PIP,AMI,VAN,CIP	BSI, disseminated	Blood, stool, TAS	Blood culture, GM positive	P: PSZ T: L-AMB + VRZ E: CFGN	ns	24 days	Died
		
		M	70	AML	CVC, digestive decontamination (GEN, COL), PIP,AMI,VAN,CIP	BSI, pulmonary	Blood	Blood culture,	E: CFGN	ns	4 days	Died
		
		F	63	AML	CVC, digestive decontamination (GEN, COL), PIP,AMI,VAN,CIP	BSI, disseminated	Blood, stool, TAS	Blood culture	E: CFGN T: L-AMB + VORI	ns	6 days (CFGN) 10 days (L-AMB + VRZ)	Died

Del Principe et al., 2016	Italy	F	36	AML	CVC, cytarabine, neutropenia, PIP-Taz, MER	Pulmonary, cholecystitis, hepatosplenic abscesses	Blood, CVC	Blood culture, betaG >500 pg/ml, GM negative	T: L-AMB VRZ (after discharge)	350 mg qd iv 200 mg bid oral	100 days (L-AMB) 15 days (FungiScope)	Survived
		
		F	50	MC lymphoma	CVC, cytarabine, steroids, neutropenia (<500 mm^3^)	Pneumonia, splenic infiltrates, sepsis	Blood	Blood culture, betaG >500 pg/ml, GM negative	T: L-AMB VRZ (after discharge)	200 mg qd iv 350 mg qd iv	10 days (L-AMB) 47 days (L-AMB)	Died
		
		M	21	AML	Methyl- prednisolone, neutropenia (<500 mm^3^), PIP-Taz, MER, cytarabine	Splenic abscesses	Blood, CVC	Blood culture, betaG negative, GM negative	T: L-AMB VRZ (after discharge)	200 mg qd iv 600 mg bid oral	12 days (L-AMB) 1 day (VRZ)	Survived

Vaux et al., 2014	France	F (15)^®^	63	AML (70%)	Neutropenia (<500 mm^3^; 90%), cytarabine (78.3%)	BSI (87%),	Blood, stool, BAL,	Blood culture	ns	ns	ns	24 (80%) died
		
		M (15)^®^	(mean)	ALL (20%)		pulmonary (40%),	TAS	(86.7%)				6 (20%) survived
				CLM (3.3%)		diarrhea (61.5%)						
				other (6.7%)								

Camus et al., 2014	France	M	32	AML	Cytarabine, IMI, VAN, MET IMI, VAN, MET	Sepsis, peritonitis, Hepatic lesions	Blood, stool, ascites	Blood culture, GM negative	E: CFGN T: VRZ	50 mg qd iv 300 mg qd iv 100 mg qd iv	8 days (CFGN) 35 days (VRZ iv) >270 days (VRZ po)	Survived

Favre et al., 2016	France	M	27	Aplastic anemia	CVC, neutropenia, prednisone, PIP-Taz, AMI, MER, LVX	BSI, disseminated	Blood, CVC	Blood culture	E: CFGN T: L-AMB + VRZ	50 mg qd iv 200 mg bid iv (L-AMB) 400 mg bid iv (VRZ)	2 days (CFGN) 55 days (L-AMB + VRZ)	Survived

de Almeida Júnior et al., 2016	Brazil	F	6	Hemophagocytic lymphohis- tiocytosis	Auto BMT, CVC, neutropenia				T: AMB-D T: VRZ	ns	18 days	Died

Fungiscope - 831	Turkey	F	37	AML (relapse)	Neutropenia (<500 mm^3^)	BSI	Blood	Blood culture	T: VRZ	240 mg bid iv 200 mg bid oral	8 days 6 days	Survived

Fungiscope - 1211	Israel	F	17	AML	Neutropenia (<500 mm^3^)	Disseminated (CNS, liver, spleen)		PCR (CSF)	E: L-AMB T: L-AMB T: 5FC T: VRZ	250 mg qd iv 250 mg qd iv 1000 mg 4x oral 200 mg bid iv	12 days 27 days L-AMB then 5 days L-AMB + 5FC 5 days L-AMB + 5FC 208 days L-AMB + 5FC + VRZ	Alive, ongoing therapy

Fungiscope - 1216	Spain	M	48	Lymphoma	alloHSCT, neutropenia (<500 mm^3^)	BSI, disseminated (CNS, liver, lung, spleen)	Blood	Blood culture, PCR (pleural fluid)	E: L-AMB E: VRZ T: L-AMB T: VRZ T: 5FC 2^nd^ P: PSZ T: L-AMB T: 5FC	400 mg qd iv 200 mg bid iv 400 mg qd iv 200 mg bid iv 37.5 mg 4x iv 300 mg qd tab 400 mg qd iv 37.5 mg 4x iv	3 days (2 days with VRZ) 2 days (with L-AMB) 99 (11 days with VRZ, then 31 days with 5FC) 11 days (with L-AMB) 31 days (with L-AMB) 92 days (mono) 9 days (with 5FC) 9 days (with L-AMB)	Died

Fungiscope - 604	Germany	M	55	AML (relapse)	alloHSCT (PBSC), neutropenia (<500 mm^3^), ICU	BSI	Blood	Blood culture	E: L-AMB T: VRZ	290 mg qd iv 200 mg bid po	5 days 30 days	Survived

Fungiscope - 616	Serbia	M	19	ALL (relapse)	Not neutropenic	BSI, pulmonary	Blood	Blood culture	E: CFGN T: CFGN	50 mg qd iv 50 mg qd iv	4 days 34 days	Died

Esposto et al., 2018	Italy	M (11)	ns	AML (8),	ns	BSI	Blood	Blood culture	ns	ns	ns	Ns
		
		F (6)		Hodgkin lymphoma (3)								
				aplastic anemia (2)								
		
		ns (1)		surgery (3), ns (2)								

Liu et al., 2018	China	M	10	Acute lymphocytic leukemia	Neutropenia, pancreatitis	BSI, pulmonary	Blood	Blood culture, GM 1.33, 6.03, beta-G 746 pg/ml	E: MFGN T: VRZ T: MFGN + VRZ T: MFGN + L-AMB	50 mg qd iv 150 mg iv q12h 100 mg qd iv + 100 mg iv q12h 27 mg iv qd	8 days (mono) 15 days (mono) 40 days (MFGN + VRZ) 43 days (MFGN + L-AMB)	Survived

Salgüero Fernándeza et al. 2018	Spain	M	47	Lymphoma	Neutropenia, prednisone, alloHSCT	BSI, skin	Blood, skin biopsy Brain abscess	Blood culture	T: L-AMB T: 5FC	5 mg/kg/d 37.5 mg 4x iv	60 days 60 days	Died

This study	Czechia	M	45	AML	CVC, cytarabine, neutropenia, alloHSCT, acute GvHD, cholelithiasis, cholecystectomy, biliary drainage	BSI, disseminated	Blood	Blood culture, GM 0.70 (-2 days)^∗^	T: AMB-D T: VRZ	75 mg qd iv 200 mg bid po	27 days 7 days	Died
		
		F	61	AML	CVC, cytarabine, neutropenia, chronic pancreatitis, cholelithiasis, cholecystectomy	BSI	Blood	Blood culture, GM 0.55 (+3 days)^∗^	T: AMB-D T: AMB-LC	75 mg qd iv 400 mg qd iv	15 days 6 days	Died
		
		F	63	AML	CVC, neutropenia (<500 mm^3^), cholelithiasis, cholecystectomy, cytarabine	BSI	Blood	Blood culture, GM 0.18, PCR (sequencing)	T: AMB-LC T: VRZ	400 mg qd iv 200 mg bid po	4 days 9 days	Survived
		
		F	58	AML	CVC, neutropenia (<500 mm^3^), cytarabine, alloHSCT, acute GvHD	BSI, pneumonia	Blood, rectum	Blood culture, GM 0.50 (only with 3^rd^ blood culture), PCR (sequencing)	T: AMB-D T: AMB-LC T: VRZ	50 mg qd iv 400 mg qd iv 200 mg bid po	2 days 7 days 4 days (AMB-LC + VRZ)	Died
		
		F	50	AML	CVC, cytarabine, neutropenia, autoHSCT	BSI, pneumonia	Blood, wound swab	Blood culture	T: AMB-D	50 mg qd iv	4 days	Died
		
		F	66	Lymphoma	CVC, cytarabine, neutropenia, cholelithiasis, cholecystectomy, *Candida glabrata* fungemia	BSI	Blood, bile, urine	Blood culture, GM 0.31	T: MFGN (*C. glabrata* fungemia) T: VRZ	100 mf qd iv 200 mg bid po	12 days (*C. glabrata* fungemia) 3 days (*S. clavata*)	Died

Leoni et al., 2018	Italy	M	6	Bone marrow failure	Three allo-HSCT neutropenia	BSI, renal, pulmonary/skin involvement	Blood	Blood culture	P: L-AMB T: L-AMB T: L-AMB + VRZ	2.5 mg/kg 2× a week 3.0 mg/kg/d iv 10 mg/kg/d iv + 8 mg/kg bid iv	16 9 days mono 30 days combo	Survived

*^#^ in general, all patients had hematological malignancy with anticancer and antibiotic therapy as predisposing conditions. §– number of all females/males in the study. ns – not specified. ^∗^ –/+ days before/after positive blood culture. 5FC – flucytosine, ALL – acute lymphoid leukemia, AMB-D/LC – amphotericin B deoxycholate/lipid complex, AMI – amikacin, AML – acute myeloid leukemia, ANC – absolute neutrophil count, BAL – bronchoalveolar lavage fluid, beta-G – beta-D-glucan, BSI – blood-stream infection, CFGN – caspofungin, CML – chronic myeloid leukemia, CIP – ciprofloxacin, CNS – central nervous system, COL – colistin, CVC – central venous catheter, FLZ – fluconazole, F/M – female/male, GEN – gentamicin, GM – galactomannan, GvHD – graft vs. host disease, HSCT – hematopoietic stem cell transplantation, ICU – intensive care unit, IMI – imipenem, L-AMB – liposomal amphotericin B, LVX – levofloxacin, MC – mantle cell, MER – meropenem, MET – metronidazole, MFGN – micafungin, P/E/T – prophylactic/empirical/targeted therapy, PIP + TAZ (piperacillin + tazobactam), PSZ – posaconazole, TAS – tracheal aspirate, VAN – vancomycin, VRZ – voriconazole.*

## Discussion

*Saprochaete clavata* together with the *Galactomyces*, *Magnusiomyces*, and *Trichosporon* species represent rare human pathogenic fungi of heterogeneous origin, which share production of arthroconidia. *S. clavata* is almost exclusively confined to systemic, life-threatening infections while the clinical presentation of infections caused by other arthroconidial fungi range from superficial (*Trichosporon* spp.), mucosal (*Galactomyces candidus*), allergic (*Trichosporon* pneumonitis) to systemic forms (*T. asahii, M. capitatus*, and *G. candidus*) (Girmenia et al., 2005; Henrich et al., 2009; Bonifaz et al., 2010; Vaux et al., 2014; de Almeida Júnior and Hennequin, 2016; Durán Graeff et al., 2017; Esposto et al., 2018; Leoni et al., 2018; Salgüero Fernández et al., 2018). AML is the leading underlying condition for systemic infections caused by *S. clavata* such as for other arthroconidial yeasts (Girmenia et al., 2005; Henrich et al., 2009; Camus et al., 2014; de Almeida Júnior and Hennequin, 2016).

All epidemiological aspects associated with *S. clavata* are not fully understood. Numbers of isolates of arthroconidial fungi obtained in our hospital during the period of 1995 to 2017 showed a noticeable fluctuation, which corresponded with similar course of fungemia outbreak in the French hospitals (Figure 3 in Vaux et al., 2014). That can suggest influence of some unknown epidemiological factor(s). All arthroconidial fungi are ubiquitous in nature but *Trichosporon* infections are more frequently described in the United States, while *M. capitatus* prevails in the Mediterranean area (Italy, France, Spain, Turkey, Greece, Tunisia, Israel, Libya, FungiScope^®^) (Schiemann et al., 1998; Gadea et al., 2004; Christakis et al., 2005; Girmenia et al., 2005; García-Ruiz et al., 2013; Vaux et al., 2014; Trabelsi et al., 2015; Del Principe et al., 2016; Durán Graeff et al., 2017; Esposto et al., 2018; Leoni et al., 2018; Salgüero Fernández et al., 2018). We found no correlation between temperature in the Czechia and in Eastern Bohemia and the number of isolated *S. clavata* strains during the follow-up period (data not shown).

As regards potential sources of these fungi, main suspicion falls on in-house environment (dishwasher) and food, especially milk and dairy products (Bouakline et al., 2000; Gurgui et al., 2011; Zalar et al., 2011; Vaux et al., 2014; Banjara et al., 2015; Gouba and Drancourt, 2015). It is worth mentioning interpersonal transmission among hospitalized patients as reported during the French outbreak and the potential of fly-to-human transmission as suggested by the positive *S. clavata* isolates from *Drosophila* flies (Pimenta et al., 2009; Vaux et al., 2014). In line with the reports on other arthroconidial fungi, the respiratory tract seems to be the main ecological niche colonized by *S. clavata* in debilitated patients, whereas the intestine and/or urogenital tract may be less relevant (Figure 6). Metagenomic studies have not revealed *S. clavata* in human microbiota in contrast to the species of *Galactomyces* and *Trichosporon*, which are part of the gut microbiome and together with *Candida, Malassezia*, and sporulating molds constitute core gut mycobiota (Gouba et al., 2014; Hallen-Adams and Suhr, 2017; Auchtung et al., 2018; Li et al., 2018).

To date, little is known about the virulence mechanisms of *S. clavata*. There is no data about biofilm production of *S. clavata*, only indirect suggestions based on a close relation between the presence of CVC and a positive blood culture (this study, Girmenia et al., 2005; Camus et al., 2014; Picard et al., 2014; Vaux et al., 2014; Del Principe et al., 2016). Compared to *T. asahii*, *S. clavata* is more genetically monomorphic (Sun et al., 2012; Vaux et al., 2014). Two main clades (A and B) of *S. clavata* were identified during the French outbreak (Vaux et al., 2014). The clinical significance of both clades was similar in most characteristics, including their susceptibility to antifungal drugs. Although clade A exhibited lower virulence expressed by longer survival of experimentally infected mice, it was responsible for most cases of the French outbreak (Vaux et al., 2014). That indicates higher human-to-human transmissibility of the clade A or its better adaptability to unknown environment (source), which can be responsible for an increased exposure of vulnerable patients to this clade (Vaux et al., 2014).

Clinically, *S. clavata* infections are difficult to distinguish from *M. capitatus* infections and the majority of other invasive mycoses. No reliable diagnostic tests are available and thus, in the absence of any specific signs and symptoms, positive blood cultivation remains indicative for this mycosis. It is difficult to establish an early diagnosis, which increases the likelihood for the optimal timing of antifungal treatment before the development of advanced and more difficult-to-control stage of the infection. There is no surprise that the mortality rate was extremely high and reached 66.6% in our patients; that was comparable to overall mortality of other reported cases (Table 4). In this way, blood culture positivity seems to represent not only diagnostic but also a poor prognostic factor.

Apart from blood culture, antigen detection can be useful in diagnosis of arthroconidial fungi because they share a cross-reactivity with cryptococcal glucuronoxylomannan (*T. asahii*, *M. capitatus*), *Aspergillus* galactomannan (*G. candidus*, *M. capitatus*), and β-D-glucan (Odabasi et al., 2006; Bonini et al., 2008; Liao et al., 2012a; Nakase et al., 2012; Trabelsi et al., 2015; de Almeida Júnior and Hennequin, 2016; Del Principe et al., 2016). In our patients, three out of five (the sixth not tested) had galactomannan index values from 0.5 to 0.7 (the other two ≤ 0.3) at the time of diagnosis of *S. clavata* fungemia (Table 4). Available data from other studies showed a lower sensitivity of the galactomannan test and questioned its practical use (Picard et al., 2014; Del Principe et al., 2016). In an Italian study, positive β-D-glucan test results were documented in two out of three patients (Del Principe et al., 2016). To date, the experience with the methods in *S. clavata* infection is little but promising results support further investigation of their clinical usefulness.

Culture-dependent identification of *Galactomyces, Saprochaete*, and *Trichosporon* is limited to AuxaColor (BioRad), API ID32C (BioMérieux) or VITEK 2 system (ID-YST card; BioMérieux). Unfortunately, none of the systems covers *S. clavata*. In general, the accuracy of identification of arthroconidial yeasts by these methods is not reliable (Posteraro et al., 2015). The use of phenotypic tests may be a source of misidentification, especially when cellobiose assimilation is missing (Smith and Poot, 1998; Desnos-Ollivier et al., 2014). Desnos-Ollivier et al. (2014) described about 15% of *S. clavata* strains that did not assimilate cellobiose. Hence, such “cellobiose-negative *M. capitatus*” strains may have escaped our attention in the past. Recently, the MALDI-TOF mass spectrometry (Biotyper 3.0) has displayed the most promising laboratory tool for determination of and discrimination between arthroconidial fungi, including *S. clavata*, even though reliability varies (Seyfarth et al., 2012; Kolecka et al., 2013). ITS, 18S rRNA or protein-coding loci (e.g., *Rbp2*) sequencing may be a reasonable approach to confirm results of other methods (this study, Desnos-Ollivier et al., 2014; Durán Graeff et al., 2017; Kaplan et al., 2017).

The role of antifungal susceptibility testing in the management of infections caused by arthroconidial fungi is controversial because of lack of standardized methods. Our MICs were influenced by changing methodologies during the follow-up period (Etest^®^, YeastOne^TM^), but most of them were in line with the results of other studies (Tables 2, 3). The inhibition zones corresponded well with the MICs in case of fluconazole, voriconazole, posaconazole, flucytosine, and echinocandins and disk test may serve as a tentative method for surveillance of *S. clavata* isolates. As no breakpoints and epidemiological cut-off are defined for *S. clavata* yet, interpretation of the susceptibility test results should be done with caution. One has to take into account the clinical form and course of the infection, the pharmacological profile of a given drug or drug formulation, and the presence of risk and predisposing factors in a patient (Arendrup et al., 2014).

Invasive infections caused by arthroconidial fungi typically manifest as fungemia with a tendency to disseminate in immunocompromised patients. They are characterized by a relatively high blood recovery rate and the involvement of different visceral organs such as the lungs, spleen and liver (Girmenia et al., 2005; Vaux et al., 2014; Cornely et al., 2015; Durán Graeff et al., 2017). Our *S. clavata* patients displayed no pulmonary symptoms, even when one patient (No. 5) was positive for bioptic sample of lungs (Table 1). This is in contrast to frequently reported findings in more than half of the French outbreak patients (Vaux et al., 2014). On the other hand, two thirds of our patients have experienced cholelithiasis or cholecystitis, which has been mentioned previously in only one female patient with *S. clavata* infection (Del Principe et al., 2016). That could be due to a relative lack of primary bile salts as a result of gallstone formation and their lower availability for the intestinal microbiota, which converts them to secondary salts with antimicrobial effect on some bacteria and also on *C. albicans* (Guinan et al., 2018; Kelly et al., 2019). Alternatively, it may be the result of antibiotic therapy or cholecystectomy that can alter composition of transformation microbiota and indirectly interfere with the production of secondary salt (Theriot et al., 2016; Wang et al., 2018). Microbiota connection is supported with the digestive tract decontamination (gentamicin and/or colistin) to which more than half of French patients have been exposed and suffered from diarrhea (Vaux et al., 2014). Another risk factor in *S. clavata* infection is anticancer drug cytosine arabinoside (cytarabine) (Stentoft, 1990; Camus et al., 2014; Picard et al., 2014; Vaux et al., 2014; Del Principe et al., 2016) with specific effect on the neutrophil count and mucosal integrity. Preferential use of more aggressive regimens of cytarabine (≥2000 mg/m^2^ twice daily) in recent years could contribute to *S. clavata* infection, like in case of five of our patients (Willemze et al., 2014).

A relatively high MIC of fluconazole (≥4 mg/l) in strains isolated from our patients suggested that the prophylactic treatment with the triazole drug could represent a selective pressure for *S. clavata* overgrowth. That is supported with the reports on development of breakthrough infections caused by arthroconidial yeasts in immunocompromised patients on fluconazole or echinocandin prophylaxis or empirical regimen (Bonini et al., 2008; Schuermans et al., 2011; Liao et al., 2012b; Durán Graeff et al., 2017).

Voriconazole remains the drug of choice for *S. clavata* infections despite not all strains display optimal *in vitro* susceptibility results (see Patient No. 2, Table 1). This is in line with the recommendation from a panel of experts (Arendrup et al., 2014). On the other hand, liposomal amphotericin B may be an effective alternative; all three Italian patients responded to liposomal amphotericin B and two of them survived (the third died of another cause) (Del Principe et al., 2016). The use of combination therapy remains controversial. Voriconazole and liposomal amphotericin B have provided mixed successes. Adding flucytosine to those drugs as suggested by Lacroix’s report and supported *in vitro* data could represent a potentially useful therapeutic modality for both (Tables 2–4) (Lacroix et al., 2007; Picard et al., 2014; Favre et al., 2016; Leoni et al., 2018; Liu et al., 2018). There are limited data about the therapeutic usefulness of posaconazole and isavuconazole (Miceli and Kauffman, 2015; Brunetti et al., 2016). Although the spectrum of activity of these antifungal drugs includes arthroconidial fungi, their MICs suggest that both drugs could be slightly less active on *S. clavata* than voriconazole, maybe, due to a lack of *in vivo* fungicidal activity and/or inadequate pharmacokinetics (Walsh et al., 1990; Girmenia et al., 2014; Pfaller et al., 2015; Durán Graeff et al., 2017; Esposto et al., 2018; Desnos-Ollivier et al., 2019). This may follow from variable host liver metabolizer status like in voriconazole (*CYP2C19* gene polymorphism) or problematic bioavailability of oral suspension of posaconazole even when the latter problem can be overcome by new formulation of delayed release tablets (Owusu Obeng et al., 2014; Yi et al., 2017; Mason et al., 2019).

The two main pillars in successful management of infections caused by *S. clavata* are the early administration of antifungal drugs and the control of underlying conditions. While antifungal can safe life for a limited period of time, long-term survival is dependent on the recovery of the underlying hematological disease or neutropenia (Camus et al., 2014; Picard et al., 2014; Del Principe et al., 2016). The only of our six patient who survived achieved a complete hematopoietic regeneration and presented fewer risk factors (shorter period of deep neutropenia, no urinary catheter, no nasogastric tube, and no parenteral nutrition) with less severe symptomatology (lack of septic shock with MODS) (Table 1).

Recovery of *S. clavata* from the blood manifests dissemination stage of life-threatening infection and underlines the urgent need to move the timing of the institution of antifungal therapy before positivity of the blood culture. That supports empirical approach to the therapy using stratification of patients and to start initial treatment based on presence or the accumulation of risk factors, urgency of clinical situation, and availability of other laboratory and clinical data (antigen detection, imaging techniques, previous microbiological findings), including response to current therapy. Therefore, management of *S. clavata* infections is complex that requires close cooperation between the clinicians, microbiologists and epidemiologists.

*Saprochaete clavata* represents an emerging opportunistic fungal pathogen closely associated with AML. Most of the clinical and epidemiological characteristics overlap with the infections caused by other arthroconidial fungi, especially *M. capitatus* and *T. asahii.* Primary source of *S. clavata* is unknown but this yeast is able to colonize humans and under favorable conditions, such as deep and long immunosuppression, to overcome debilitated defense mechanisms and cause life-threatening infection. The prognosis of these invasive infections is generally poor due to lack of the specific clinical signs and symptoms, reliable diagnostic methods, and a limited efficacy of available antifungal drugs. The diagnosis of *S. clavata* infections is usually based on positivity of blood culture; detection of beta-D-glucan or *Aspergillus* galactomannan can be helpful. The optimal treatment has not been established yet; best results are connected with the application of voriconazole or liposomal amphotericin B, but successful outcome is usually critically dependent on the recovery of underlying conditions associated with immune dysfunction or deficiency.

## Data Availability Statement

The datasets generated for this study are available on request to the corresponding author.

## Author Contributions

VB contributed conception and design of the study, analyzed and interpreted the patient and microbiological data, and wrote the manuscript. RB analyzed and interpreted the patient data regarding molecular analysis. EH analyzed and interpreted the patient data regarding the hematological disease. OC and DS reviewed the manuscript and provided FungiScope data. PŽ analyzed and interpreted the patient data regarding the hematological disease and wrote the manuscript. All authors contributed to manuscript revision, read and approved the submitted version.

## Conflict of Interest

The authors declare that the research was conducted in the absence of any commercial or financial relationships that could be construed as a potential conflict of interest.

## Footnotes

1 http://blast.ncbi.nlm.nih.gov/Blast.cgi
